# ASO Author Reflections: How Many Centers Do We Need for High-Quality Penile Cancer Surgery in Germany? An Analysis of Total Population Data from 2006 to 2016

**DOI:** 10.1245/s10434-021-10306-5

**Published:** 2021-06-16

**Authors:** Christer Groeben, Rainer Koch, Klaus Kraywinkel, Nina Buttmann-Schweiger, Martin Baunacke, Angelika Borkowetz, Christian Thomas, Johannes Huber

**Affiliations:** 1 Department of Urology, Medical Faculty Carl Gustav Carus, TU Dresden, Dresden, Germany; 2 National Center for Cancer Registry Data, Robert Koch Institute, Berlin, Germany

## Past

Penile cancer is a rare disease in Western countries. However, for tumors that have already progressed locally with vascular, corporal, or urethral invasion, mutilating surgery with partial or total amputation of the penis often is the only curative option.^1^^,^^2^ Consequently, guidelines strongly recommend referring those patients to specialized centers. However, data on recent treatment patterns for surgical management of penile cancer in Germany are lacking. Thus, we aimed to analyze trends in surgical treatment patterns for penile cancer in Germany.

## Present

We therefore assessed data from the nationwide German hospital billing database and the German cancer registry covering the years 2006 to 2016. During the investigated time span, the annual incidence of penile cancer increased by 29.8% (in absolute numbers), similar to the increase in other European countries.^3^ Consequently, the caseload increased by 35% for penile cancer surgery and by 53.3% for lymph node dissection (LND). During this development, the proportion of cases managed in hospitals with a high caseload increased by 9.8 percentage points for penile surgery and by 13.1 percentage points for LND. The increase in the LND caseload was caused mainly by increasing inguinal LND numbers while open and laparoscopic pelvic LND remained stable. Inguinal LND was performed predominantly in a radical or modified fashion, with a slight trend toward an increasing use of the sentinel technique. Assessment of the geographic distribution of cases in Germany showed extensive areas without sufficient coverage by experienced centers.

## Future

First of all, our findings demonstrated treatment patterns for surgical management of penile carcinoma in Germany using total population data covering a comparatively long period of 11 years to depict possible developments over time. The increase in caseload numbers may have been caused by the increasing proportion of older men in our society.^4^ Thus, we expect a slowly increasing caseload in the future. For rare diseases such as penile cancer, widespread guideline implementation can be problematic. Despite this, the increasing caseload numbers of invasive lymph node staging show progressive implementation of guideline recommendations.^1^^,^^5^ Adding national cancer registry data and the regional distribution of penile cancer care providers complemented these total population data to draw a more complete picture of the German situation. Nevertheless, the geographic distribution of experienced centers in Germany could be improved by respective health policymaking to provide patients with adequate treatment in their regional vicinity.
